# Seroprevalence and risk factors for *Rickettsia felis* exposure in dogs from Southeast Queensland and the Northern Territory, Australia

**DOI:** 10.1186/1756-3305-6-159

**Published:** 2013-06-03

**Authors:** Sze-Fui Hii, Mohammad Y Abdad, Steven R Kopp, John Stenos, Robert L Rees, Rebecca J Traub

**Affiliations:** 1 School of Veterinary Science, The University of Queensland, Gatton, Queensland, 4343, Australia; 2 Australian Rickettsial Reference Laboratory, Geelong, Victoria, 3220, Australia; 3 Bayer Animal Health Tingalpa, Tingalpa, Queensland, 4173, Australia

**Keywords:** Rickettsia felis, Flea-borne spotted fever, Seroprevalence, Ctenocephalides felis

## Abstract

**Background:**

The recent detection of *Rickettsia felis* DNA in dogs in Australia suggests that dogs are potential mammalian reservoir hosts for this emerging rickettsia. To date, there is no published report addressing the seroprevalence of *R. felis* in dogs in Australia.

**Methods:**

Antigens for *R. felis* were produced by inoculating confluent XTC-2 monolayer cell cultures with three pools of cat flea (*Ctenocephalides felis*) homogenates. Infection was confirmed by real-time (qPCR), conventional or nested PCRs targeting the *omp*B, *glt*A, 17 kDa and *omp*A genes. Two hundred and ninety-two dogs from Southeast Queensland and the Northern Territory were tested for the presence of *R. felis* antibodies using a microimmunofluorescence (IF) test and the seroprevalence and associated risk factors for exposure were determined using both uni- and multi-variate analyses.

**Results:**

*Rickettsia felis* was successfully isolated in cell culture from all three cat-flea pools. One hundred and forty-eight dogs (50.7%) showed seropositivity with titres ≥64 and 54 (18.5%) with titres ≥128. At antibody titres ≥64, dogs with active ectoparasite control were less likely to be seropositive to *R. felis* (OR: 2.60; 95% CI: 1.20 - 5.56).

**Conclusions:**

This first reported isolation of *R. felis* in cell culture in Australia allowed for the production of antigen for serological testing of dogs. Results of this serological testing reflects the ubiquitous exposure of dogs to *R. felis* and advocate for owner vigilance with regards to ectoparasite control on domestic pets.

## Background

A number of rickettsial species are associated with human disease in Australia. These include Queensland tick typhus caused by *R. australis*, Flinders Island spotted fever caused by *R. honei*, Australian spotted fever by *R. honei* subspecies ‘*marmionii*’’, epidemic typhus by *R. prowazekii*, murine typhus by *R. typhi*, scrub typhus by *O. tsutsugamushi* and Q fever by *Coxiella burnetti*[1]. In recent years, the ubiquitous nature and potential veterinary public health significance of *Rickettsia felis* as an emerging rickettsial zoonosis that causes flea-borne spotted fever (FSF) has become increasingly apparent [2-6]. An increasing number of human cases have being reported worldwide, and in Australia the agent was reported for the first time affecting five household members ranging in age from 4–64 years, living with flea-ridden pets in Victoria, Australia [2].

The ubiquitous nature of *R. felis* and the risk it poses to human health is largely due to the global distribution of its biological vector, the ‘cat flea’ *Ctenocephalides felis*[5,7]. Infected cat fleas have been described in over 20 countries spanning five continents, with infection rates ranging from 15% in New Zealand [8] to 81% in New Caledonia [9]. In Australia, 19.8% of flea pools collected from cats in eastern Australia [10], 36% from dogs and 33% from cats in Western Australia [11], and 48.5% from dogs in Southeast Queensland (SE QLD) and the Northern Territory (NT) (Hii *et al*., unpublished data) were demonstrated to carry *R. felis* DNA.

Although *C. felis* has been studied extensively and is a well-recognised biological vector for *R. felis,* surprisingly there is to date no consensus on the potential mammalian reservoir(s) for this emerging zoonosis. Several peri-domestic species associated with the cat flea have been implicated, including cats, dogs, opossums and rats, all of which have been naturally seropositive or molecular positive for *R. felis* infection [3,12]. In Spain, 51.1% of dogs had detectable antibodies to *R. felis*[13] supporting their role as potential reservoir hosts. On the other hand, a relatively low seroprevalence (1.4% - 13.1%), was documented in dogs from Brazil [14-16].

Recently, 9% of pound dogs in SE QLD and 2.3% of Indigenous community dogs in the NT, Australia were found to have detectable *R. felis* DNA in their blood, implying that domestic dogs were likely primary reservoir hosts for *R. felis*[17,18]. In these studies, all dogs appeared healthy, a common feature that is also usually a characteristic of reservoir hosts. To date seroepidemiological studies on rickettsial diseases involving dogs have focussed on their role as possible sentinel hosts for human rickettsioses in Australia. In 1991, 11.2% of dogs from south eastern Australia, which included coastal New South Wales, eastern coastal Victoria, Flinders Island, and the Tasmanian mainland, were found to be seropositive to *R. australis* infection [19]. A serosurvey in Launceston, Tasmania, where spotted fever group (SFG) diseases are endemic, demonstrated that 57% of dogs had been exposed to SFG rickettsiae [20]. Recently, antibodies reactive with *Coxiella burnetii* were detected in 21.8% of domestic dogs from northern Queensland [21].

In this study, we isolated *R. felis* in cell culture to allow for the production of antigen for serological assays. We aimed to determine the seroprevalence and associated risk factors for exposure to *R. felis* in dogs from previously sampled regions in Queensland and the Northern Territory in order to support earlier findings suggesting that dogs were primary mammalian reservoir hosts for this agent.

## Methods

### Sampling and PCR

Single blood samples were collected into clotting tubes from a total of 292 dogs sourced from pounds, veterinary practices in SE QLD the NT and the Clinical Pathology Laboratory (CPL) based at the School of Veterinary Science, The University of Queensland. Sera was subsequently collected from clotting tubes and stored at −80°C until analysed.

Pound dogs used for teaching purposes were sourced from the Clinical Studies Centre, School of Veterinary Science, The University of Queensland. Samples from client-owned dogs were sourced from five veterinary practices across SE QLD and one from Katherine in the NT. These dogs were presented to veterinary practices for many reasons including routine vaccination, neutering, heartworm testing, yearly health profiling and a range of illnesses. Blood and sera from the CPL were based on convenience; these samples were archived routine diagnostic specimens and would have otherwise been discarded. Following blinding for owner confidentiality, information with regards to age, sex, breed and ectoparasite control were recorded. This project was approved by the University of Queensland Animal Ethics Committee.

### Isolation of R. felis in cell culture

*Rickettsia felis* antigen was isolated using XTC-2 cell lines, courtesy of the Australian Rickettsial Reference Laboratory, Geelong, Victoria. XTC-2 cell lines were cultured in 25 cm^2^ cell culture flasks with Leibowitz-15 (L-15) (GIBCO, Rockville, MD) medium supplemented with 5% (v/v) foetal calf serum (Bovogen Biologicals, Australia), 2 mM L-glutamine and L-amino-acids (GIBCO, Rockville, MD), and 1% (v/v) tryptose phosphate (GIBCO) [22]. Cell lines were incubated at 28°C for 48–72 hours to obtain subconfluent cell monolayers.

Three pools of 20 live cat fleas, one collected from a pound dog in SE QLD and two from laboratory colonies maintained at the School of Veterinary Science, The University of Queensland were collected. These were surface sterilized by washing in 2% iodine for 3 minutes and 70% ethanol for 2 minutes, followed with a rinse in sterile distilled water. They were collected into 1.5 ml centrifuge tubes containing 100 μl culture medium and ground with sterile plastic pestles. One millilitre of culture medium containing 100 μg/ml gentamicin was added and the flea homogenate mixed. Five hundred microlitres of homogenate was transferred using a syringe filter (with a 0.45 μm membrane) into a 25 cm^2^ cell culture flask containing the XTC-2 monolayer cell lines with approximately 12 ml of the antibiotic medium. The remaining homogenate was kept at −20°C for PCR testing. The flasks were centrifuged at 250 *g* for 5 minutes at 20°C. This was followed by a 24 hour incubation, after which, the media was replaced with antibiotic-free media. The inoculated cell lines were examined daily for contamination under a tissue culture microscope. The media was changed fortnightly and screened for rickettsial infection by Diff-Quick staining (Quick Dip, Fronine Lab Supplies, Australia), qPCR and conventional PCR.

DNA of flea homogenates and inoculated cell lines was extracted using the DNeasy Blood & Tissue Kits (QIAGEN, Hilden, Germany) following the manufacturer’s protocol. All extracted DNA of fleas and cell cultures were subjected to qPCR to detect the *glt*A gene according to the previous protocol [23], with some modification. Reactions were performed in a 10 μl mixture containing Kapa Probe Fast qPCR mastermix (Kapa Biosystems), 4 pmol of each forward and reverse primers, 2 pmol of probe and 2 μl of extracted DNA. All qPCR positive DNA samples were further analysed using a single rickettsiae-specific PCR targeting partial *omp*B and 17 kDA genes, and a nested *R. felis*-specific PCR targeting *glt*A genes [17,18,24] followed by bidirectional DNA sequencing to confirm rickettsial speciation.

In addition, an *R. felis*-specific PCR was developed to amplify a 1009 bp of the *omp*A gene using newly designed primers - *omp*A-F1 5’-CGATAGTGTTACAAGTACCGG-3’ and *omp*A-R1 5’-GCATCTTCCATTAACTCAAGC-3’. PCRs were performed in a 25 μl reaction mixture containing 2 μl of DNA, 5 μl 5x PCR buffer, 200 μmol/L dNTP, 2.0 mmol/L MgCl_2_, 0.5 units of GoTaq polymerase (Promega, Madison, WI, USA), 10 pmol of each forward and reverse primer and a final volume of nuclease free water. PCRs were run at 95°C for 2 min for the initialization step, followed by 40 cycles of 95°C for 45 s, 57°C for 30 s and 72°C for 45 s with a final extension step of 72°C for 7 min. All amplified PCR products were subjected to DNA sequencing.

### Preparation of IF test slides

*Rickettsia felis* infected XTC-2 cell lines were harvested and inoculated into an uninfected monolayer of XTC-2 cell lines in the 25 cm^2^ cell culture flask. Cell lines were harvested when the infection rate of cells reached 90%, as estimated by IF and Diff-Quick staining. The infected cells with medium were centrifuged at 500 *g* for 5 minutes and the supernatant was discarded. The pellet was resuspended with sterile 1 x PBS and heat inactivated at 56°C for 30 minutes. Two microlitres of the antigen was spotted onto each of the 40 well slides, air dried, and fixed in acetone for 10 minutes. Slides were kept at 4°C until used.

### IF test

An IF was performed following a previously described protocol [20,25] with some modification. In brief, each serum sample was screened for *R. felis* antigen at 1:32 dilution in a 2% skimmed milk-PBS solution. All slides were incubated in a humid chamber at 37°C for 30 minutes, then washed with 1/10 PBS for 3 minutes and air-dried. Fluorescein isothiocyanate (FITC)-labelled goat anti-dog immunoglobulin G (Kirkegaard & Perry Laboratories, USA) was added and slides were incubated, washed, air-dried, mounted with fluorescence mounting medium (Dako, USA) and visualized under a UV microscope. Positive and negative dog sera were used as control in each reaction. Negative control serum was sourced from a dog previously tested to be non-reactive to *R. felis, R. australis, R. honei, R. typhi, R. conorii* and *R. rickettsii*. Positive control serum was sourced from a dog tested to be reactive only to *R. felis* independently by the Australian Rickettsial Reference Laboratory.

All sera showing a positive reaction at 1:32 were subjected to serial doubling dilution until an end-point was obtained. Discordant samples were read by a second examiner independently to confirm endpoint reactivity. Sera with titres of 1:64 or greater were considered positive, as previously described [13,15,19].

### Statistics

Statistical calculations were conducted using SPSS version 20.0 software (SPSS Inc., Chicago, IL, USA). The association between *R. felis* seropositivity (at titres ≥64 and ≥128) and putative risk factors (age, sex, breed, ectoparasite treatment status and ownership status) were evaluated in the univariable analysis using logistic regression models. Odds ratios and their 95% confidence intervals were reported for each risk factor. Exact tests were used to evaluate the association of dichotomous risk factors with the presence of *R. felis* antibodies. Overall *P*-values for risk factors with more than two categories were assessed using joint-significance hypothesis tests.

After checking for collinearity, variables significant at *P* ≤0.2 and with sufficient numbers (n > 10) in the univariable analysis were considered eligible for inclusion in the multiple logistic regression analysis [26,27]. Backward elimination was used as a model building approach and risk factors were dropped from the multivariable model until all risk factors in the model were statistically significant at P < 0.05. [27].

## Results

### Antigen production

*Rickettsia felis* was successfully isolated from all three inoculated XTC-2 cell lines as detected by qPCR, single (*omp*B and 17 kDa) and nested PCR (*glt*A), and Diff-Quick staining at 4 weeks post incubation. Partial *omp*A gene of *R. felis* was also amplified in all infected cell lines. The isolation of *R. felis* enabled the production of antigen for IF testing.

A total of 292 dog sera were collected from December 2009 to December 2012–185 from SE QLD and 107 from NT. Of these, 100 were pound dogs, 162 were client-owned dogs sourced from referral practices and 30 were convenience samples from the CPL. Of the CPL sourced samples, ownership status was confirmed in 18 dogs. In total, 180 dogs were client-owned.

There were 142 purebred dogs, 147 of mixed breed and 3 were of unknown breed. Most (66.9%) dogs were adults (1–10 year), followed by young dogs (<1 year) (20.2%) and geriatrics (>10 years) (12.9%). One hundred and forty three were male (49.7%), 145 were female (50.3%) and 4 were of unspecified sex. Ectoparasite control status was only available for 48 client-owned dogs. Of these, 42 dogs were subjected to ectoparasite control. Consultation with staff revealed that pound dogs had not received active ectoparasite control.

A total of 148/292 (50.7%) and 54/292 (18.5%) dogs were seropositive for *R. felis* with antibody titres of ≥64 and ≥128 respectively (Table 1). Of these, 94 had an antibody titre of 1:64, 42 an antibody titre of 1:128, 10 an antibody titre of 1:256, 1 an antibody titre of 1:512 and 1 an antibody titre of 1:8192.

**Table 1:** **Univariate analysis of risk factors and their association with *R. felis *seropositivity in dogs at antibody titres ≥64 and ≥128**

**Variable surveyed**	**No of sera available**	**Antibody titre ≥64**			**Antibody titre ≥128**		
		**No of seropositive dogs (%; 95% CI)**	**OR; 95% CI**	***P *value**	**No of positive dogs (%)**	**OR; 95% CI**	***P *value**
**Total sera in the study**	**292**	**148 (50.7%)**	**0.45 - 0.56**		**54 (18.5%)**	**0.14 - 0.23**	
**Location**				**0.360**			**0.947**
SE QLD dogs	185	90 (48.6%)	Reference		34 (18.4%)	Reference	
NT dogs	107	58 (54.2%)	1.25; 0.78 – 2.01		20 (18.7%)	1.02; 0.55 – 1.88	
**Source**				**0.510**			**0.221**
Client-owned	180	88 (48.9%)	Reference		29 (16.1%)	Reference	
Pound	100	53 (53.0%; )	1.18; 0.72 – 1.87		22 (22%)	1.47; 0.79, 2.72	
**Status of active ectoparasite control**				**0.014**			**0.106**
Active ectoparasite control	42	12 (28.6%)	Reference		4 (9.5%)	Reference	
No active ectoparasite control	106	52 (49.1%)	2.60; 1.20 - 5.56		22 (20.8%)	2.49; 0.80, 7.69	
**Breed**				**0.265**			**0.286**
Purebred	142	67 (47.2%)	Reference		23 (16.2%)	Reference	
Mixed breed	147	79 (53.7%)	1.30; 0.76 - 2.51		31 (21.1%)	1.38; 0.76, 2.51	
**Age**				**0.298**			**0.128**
Young (<1 year)	58	25 (43.1%)	Reference		7 (12.7%)	Reference	
Adult (1 – 10 year)	192	101 (52.6%)	1.37; 0.76 – 2.46		41 (21.4%)	1.98; 0.84 – 4.69,	0.121
Geriatric (>10year)	37	18 (48.6)	1.17; 0.51 – 2.67		4 (10.8%)	0.88; 0.24 – 3.25	0.852
**Gender**				**0.638**			**0.414**
Male	143	70 (49.0%)	Reference		29 (20.3%)	1.28; 0.71, 2.33	
Female	145	75 (51.7%)	1.12; 0.70 – 1.77		24 (16.6%)	Reference	
**Desexing status**				**0.032**			**0.062**
Neutered	111	48	Reference		16 (14.4%)	Reference	
Intact	111	64	1.79; 1.05 – 3.04		27 (24.3%)	1.91; 0.96 – 3.78	

CI, confidence interval.

OR, Odds ratio.

Of the seven risk factors assessed in the univariable model, only ectoparasite prevention and desexing status were included in the multivariable logistic regression analysis. Cross-tabulation of desexing status at antibody titres of ≥ 64 stratified for gender, indicated that 20/55 (36.4%) of neutered females were seropositive for *R. felis* while 34/51 (66.7%) of intact females were seropositive (*P* = 0.002). This relationship was not significant for males (P = 0.574). Hence, gender was forced into the multivariable model to explore the interaction between gender and desexing status. However, this interaction term was not significant in the multivariable model. The analysis revealed that dogs receiving no ectoparasite control (odds ratio 2.6, 95% CI: 1.20 – 5.56, *P* = 0.014) were more likely to have antibodies to *R. felis* at titres of ≥ 64. No risk factors were associated with *R. felis* antibody titres of ≥ 128 at P < 0.05.

## Discussion

This study represents the first isolation of *R. felis* in cell culture from cat fleas in Australia. This pathogenic agent is an obligate intracellular bacteria which requires nucleated eukaryotic cells to grow [28], and grows best at temperatures under 32°C [3]. XTC-2 cell lines are derived from *Xenopus laevis*, a South African clawed toad, which grows at 28°C and is suited to support the growth of *R. felis* at optimum levels. In contrast, the optimal growing temperatures for typhus group (35°C) and spotted fever group (32°C) rickettsiae are higher [29,30].

Previous isolation of *R. felis* in XTC-2 cell lines was attempted using the shell vial centrifugation technique [22]. This technique is sensitive and frequently utilised for isolation of agents from clinical specimens [31,32] that contain a low burden of microorganisms. However, it is laborious, requires expertise and is not suitable for downstream production of antigen for serological assays. In this study, conventional cell culture was carried out utilising cell culture flasks to enable production of *R. felis* antigens in large amounts. *Rickettsia felis* has also been reportedly successfully cultivated in vertebrate and arthropod cell lines, including Vero cells, L929, ISE6 and C6/36 [22,33-35].

Our study represents the first to provide serological evidence for *R. felis* exposure in dogs in Australia. The high seroprevalence (50.7%) is in agreement with a study conducted in Spain, where 51.1% of dogs were reported as exposed to this agent [13]. The high seroprevalence of *R. felis* in dogs in the present study was not unexpected. The cat flea, *C. felis,* is known to be the most common ectoparasite and dominant flea infesting dogs in Australia and its wide geographical distribution across the country [11,36] suggests that the seroprevalence of *R. felis* reported in this study could be a representation of most populated areas of Australia. We found no significant difference in seropositivity between dogs located in SE QLD and NT despite the variation in climate. This suggests that dogs from these two regions have been equally exposed to *R. felis*, which is in turn likely attributable to frequent exposure to cat fleas. However, flea infestation in dogs in the current study was not evaluated, hence an association with the presence of *R. felis* antibodies could not be confirmed.

Besides fleas, DNA of *R. felis* has also been isolated from the brown dog tick, *Rhipicephalus sanguineus*[37]. This tick is highly prevalent in dogs in the NT due to its preference for the humid warm tropics with relative humidity of 60%-90% and temperatures of 20°C −30°C [38]. Whether this tick species acts as a true biological vector as opposed to simply being an incidental mechanical vector remains uncertain at this time.

Serological cross-reactivity among *Rickettsia* spp is common. *R. felis* antibodies have been known to be more reactive to *R. typhi* from the typhus group, than to the spotted fever group [2,7,39,40]. Moreover, a recent serosurvey study in Spain showed dogs that were positive for *R. felis* antibodies did not necessarily cross-react with *R. typhi*, with prevalences of 9.7% and 51.1% respectively [13]. A seroepidemiological study of *R. felis, R. typhi* and *R. conorii* infection in humans in Spain also demonstrated low levels of cross-reaction between *R. felis* and *R. typhi* or *R. conorii*[41]. These findings might suggest the possibility of high specificity of *R. felis* serological tests.

The current study highlights the importance of flea control in pets by demonstrating a significant association between active ectoparasite control and the absence of *R. felis* exposure. Although it is not statistically significant in the multivariable model, intact female animals in the current study showed higher seroprevalence of *R. felis* compared to neutered dogs*,* suggestive of possible association with gonadal hormonal factors that might influence the outcome of an infection [42]. Sex-associated behaviour such as roaming in intact males may predispose them to wider exposure to fleas and the pathogens they carry. This phenomenon has been observed in a number of studies whereby neutering decreased the prevalence of both endoparasites and tick-borne diseases in dogs [42-45].

The high seroprevalence in dogs in the present study, the detection of *R. felis* DNA in dog blood [17] and high infection rates in cat fleas sourced from dogs [11] support the role of dogs as potential reservoir hosts for this zoonosis [46]. Previous studies have demonstrated infection with rickettsial spotted fever in humans positively associated with owning or contacting dogs [47,48]. In Spain, seropositivity was associated with humans who had contact with domestic animals compared to farm and wild animals [49]. A dog whose owners were infected with FSF was also found to be infected by the same agent [4]. This study further provides evidence of the risks this emerging zoonosis poses, especially to companion animal owners and their families.

## Conclusion

This study reports the first isolation of *R. felis* from *C. felis* in cell culture in Australia. This study reflects the natural ubiquitous exposure of dogs to *R. felis* in tropical and subtropical parts of northern and eastern Australia and advocates for owner vigilance with regards to ectoparasite control on domestic pets.

## Abbreviations

qPCR: Real-time Polymerase chain reaction; SE QLD: Southeast Queensland; NT: Northern Territory; FSF: flea-borne spotted fever; SFG: spotted fever group; CPL: Clinical Pathology Laboratory; IF: microimmunofluorescence test.

## Competing interests

The authors declare that they have no competing interests.

## Authors’ contributions

SFH carried out the laboratory work, data analysis, intellectual interpretation and writing of the manuscript. MYA supervised the study, carried out the laboratory work, intellectual interpretation and critical revision of the manuscript for publication. RJT designed the study project, supervised the study, and was involved in intellectual interpretation and critical revision of the manuscript for publication. SRK supervised the study and was involved in intellectual interpretation and critical revision of the manuscript for publication. JS and RLR revised the article critically for important intellectual content. All authors read and approved the final version of the manuscript.
